# Device therapies in cardiorenal syndrome: an expert review

**DOI:** 10.1093/eschf/xvag195

**Published:** 2026-08-18

**Authors:** Jeroen Dauw, Guillaume Baudry, Marta Cobo Marcos, Mateusz Guzik, Juan Carlos Lopez, Biykem Bozkurt, Gracjan Iwanek, Masatake Kobayashi, Òscar Miró, Peder Langeland Myhre, Alberto Palazzuoli, Jeffrey M Testani, Marco Metra, Piotr Ponikowski, Wilfried Mullens, Jan Biegus

**Affiliations:** Cardiovascular Center Aalst, AZORG Hospital, Moorselbaan 164, Aalst 9300, Belgium; INSERM, Centre Investigations Cliniques 1433, Inserm 1116 and INI-CRCT (Cardiovascular and Renal Clinical Trialists) F-CRIN Network, Université de Lorraine, CHRU de Nancy, Nancy, France; REICATRA, Recherche et Enseignement en IC Avancée, Transplantation, Assistance, Vandœuvre-lès-Nancy, France; Cardiology Department, Hospital Universitario Puerta de Hierro-Majadahonda, Madrid, Spain; Centro de Investigación Biomédica en Red (CIBER Cardiovascular), Madrid, Spain; Department of Cardiology, Clinical Department of Intensive Cardiac Care, Faculty of Medicine, Institute of Heart Diseases, Wroclaw Medical University, Wroclaw, Poland; Cardiology Department, Hospital Universitario Puerta de Hierro-Majadahonda, Madrid, Spain; Centro de Investigación Biomédica en Red (CIBER Cardiovascular), Madrid, Spain; Cardiology Section, Winters Center for Heart Failure, Baylor College of Medicine, Houston, TX, USA; Department of Cardiology, Clinical Department of Intensive Cardiac Care, Faculty of Medicine, Institute of Heart Diseases, Wroclaw Medical University, Wroclaw, Poland; Department of Cardiology, Tokyo Medical University, Tokyo, Japan; Emergency Department, Hospital Clinic, IDIBAPS, University of Barcelona, Barcelona, Spain; Department of Cardiology, Oslo University Hospital Rikshospitalet, Oslo, Norway; Department of Cardiology, Akershus University Hospital, Lørenskog, Norway; Institute of Clinical Medicine, University of Oslo, Oslo, Norway; Cardiovascular Diseases Unit, Cardio Thoracic and Vascular Department, S. Maria Alle Scotte Hospital, University of Siena, Siena, Italy; Section of Cardiovascular Medicine, Yale University, New Haven, CT, USA; Department of Cardiology, Vita-Salute San Raffaele University, IRCCS San Raffaele Hospital, Milan, Italy; Department of Cardiology, Clinical Department of Intensive Cardiac Care, Faculty of Medicine, Institute of Heart Diseases, Wroclaw Medical University, Wroclaw, Poland; Department of Cardiology, Ziekenhuis Oost-Limburg, Genk, Belgium; Biomedical Research Institute, Faculty of Medicine and Life Sciences, LCRC, UHasselt, Diepenbeek, Belgium; Department of Cardiology, Clinical Department of Intensive Cardiac Care, Faculty of Medicine, Institute of Heart Diseases, Wroclaw Medical University, Wroclaw, Poland

**Keywords:** Cardiorenal syndrome, Acute heart failure, Decongestion, Device therapy, Diuretic resistance

## Abstract

Managing cardiorenal syndrome in acute decompensated heart failure remains a major challenge in contemporary cardiology. While pharmacological decongestion with diuretics is the cornerstone of therapy and is effective in most patients, worsening renal function and diuretic resistance continue to pose challenges in selected cases. Device-based therapies are increasingly explored to target key pathophysiological mechanisms underlying cardiorenal dysfunction. This expert review builds on a conceptual framework for device-based therapies. Rather than cataloguing individual technologies, we apply a functional framework that classifies devices according to their primary mode of action: reduction of venous or lymphatic congestion (‘pullers’), augmentation of arterial perfusion (‘pushers’), and direct removal of sodium and/or fluid (‘removers’). For each category, we integrate pathophysiological rationale with available clinical evidence, highlighting the predominance of early-phase physiological evidence and the need for randomized studies demonstrating benefit beyond optimized contemporary medical care.

## Graphical Abstract

**Graphical Abstract:**
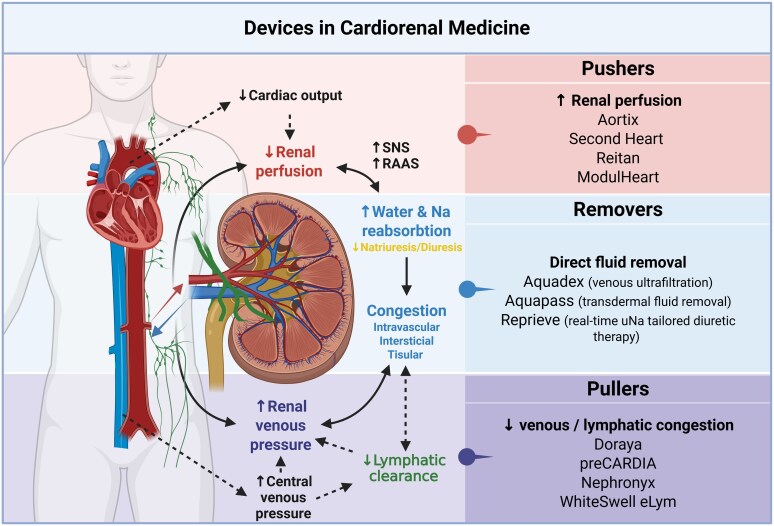
Devices in cardiorenal syndrome. The figure illustrates the different types of devices that can address the pathophysiology of cardiorenal syndrome in acute heart failure. Dotted arrows indicate direct haemodynamic alterations in heart failure. Solid arrows indicate renal alterations in the context of heart failure. Dotted lines indicate the contribution of devices in cardiorenal medicine. Sodium (Na), renin–angiotensin–aldosterone system (RAAS), sympathetic nervous system (SNS), spot urinary sodium (uNa).

## Introduction

Heart failure (HF) and chronic kidney disease (CKD) frequently coexist, and their interaction contributes to a higher risk of congestion, recurrent hospitalizations and renal dysfunction.^1,2^ In acute decompensated heart failure (ADHF), worsening renal function (WRF) often complicates the clinical course.^1^ While a rise in creatinine during decongestion often does not reflect structural kidney injury,^3^ it may coincide with a state of diuretic resistance or haemodynamic instability that limits the effectiveness of pharmacological therapy.^4^ In addition, definitions of worsening renal function and diuretic resistance are not standardized and may differ between acute and chronic settings, contributing to variability in patient characterization and treatment strategies.

Although most patients can be effectively decongested with optimized pharmacological therapy, a subset remains difficult to manage because of persistent congestion, haemodynamic instability, or apparent diuretic resistance. For these patients, a variety of devices are currently in early-stage clinical or pre-clinical development and aim to target the cardiorenal axis to facilitate decongestion.

The objective of this review is to provide a structured and mechanistic overview of device-based therapies in the context of cardiorenal syndrome. This review categorizes these devices based on their primary physiological target: those that reduce renal venous hypertension (‘pullers’), those that augment renal arterial flow (‘pushers’), and those designed for the direct mechanical removal of sodium and fluid (‘removers’). By integrating available clinical evidence with underlying pathophysiological rationale, this review aims to clarify the potential role, limitations, and future directions of device-based strategies in HF complicated by cardiorenal dysfunction. Of note, short- and long-term mechanical circulatory support devices, such as left ventricular assist devices (LVAD) and extracorporeal membrane oxygenation (ECMO), are beyond the scope of this review as they primarily aim to improve haemodynamics in general, rather than tackling cardiorenal syndrome specifically. They have been extensively described elsewhere.^5^

## Pathophysiology

Renal dysfunction during ADHF arises from a complex interplay among congestion, altered haemodynamics, and adaptive renal responses (*Graphical Abstract*)^6–12^ In this context, alterations in venous capacitance and venous tone, including changes in stressed blood volume, contribute to fluid redistribution and increased cardiac filling pressures, even in the absence of major changes in total blood volume.^13^ Venous congestion is increasingly recognized as a major contributor to cardiorenal dysfunction, particularly through elevations in right-sided cardiac filling pressures.^14,15^ Increased right atrial and central venous pressures are directly transmitted to the renal venous circulation, potentially leading to a reduction in the effective renal perfusion gradient despite preserved systemic arterial pressure. Under these conditions, impaired venous drainage and rising interstitial pressure may constrain renal blood flow and filtration, even in the absence of major reductions in arterial pressure or cardiac output, with direct theoretical consequences for natriuresis and diuretic response.^16^ These changes may impair sodium excretion, contributing to the dissociation between estimated glomerular filtration rate, cardiac output, and diuretic response.^17^

Elevated intra-abdominal pressure can further impair renal venous drainage and reduce effective filtration pressure, independent of systemic haemodynamics^18–20^ At the same time, the lymphatic system, which normally facilitates the clearance of interstitial fluid, may become overwhelmed in the setting of sustained venous hypertension, limiting its capacity to buffer rising tissue pressures^21–25^

In addition to venous congestion, impaired renal perfusion may contribute to renal dysfunction during decompensation. Renal function is more closely related to effective arterial perfusion pressure than to cardiac output itself, and autoregulatory mechanisms can preserve renal blood flow and glomerular filtration across a broad range of haemodynamic conditions.^26^ Consequently, low cardiac output, although present in a subset of patients with ADHF,^27^ does not necessarily translate into impaired renal function.^26^ Nevertheless, when effective renal perfusion becomes critically compromised and autoregulatory capacity is exceeded, reduced arterial perfusion may contribute to worsening renal function, neurohormonal activation, and impaired sodium handling.^8,28^ The relative contribution of congestion and impaired perfusion varies across patients and over time,^8,28^ contributing to the marked heterogeneity in renal response and treatment effects observed in clinical practice.^4^

Beyond haemodynamic abnormalities, alterations in tubular sodium handling represent a third component of cardiorenal dysfunction. Neurohormonal activation, reduced distal sodium delivery, and structural adaptation of the nephron increase sodium avidity and blunt the natriuretic response to loop diuretics.^6,10,28^ This dissociation between administered diuretic therapy and natriuretic response is a defining feature of diuretic resistance.^29^

Renal dysfunction and impaired decongestion arise from varying combinations of venous congestion, altered perfusion, and tubular adaptation, often superimposed on pre-existing structural kidney disease^4,30–32^ This provides the rationale for complementing pharmacological decongestion with device-based strategies targeting these specific aspects of pathophysiology.

## Pullers

**Figure 1:**
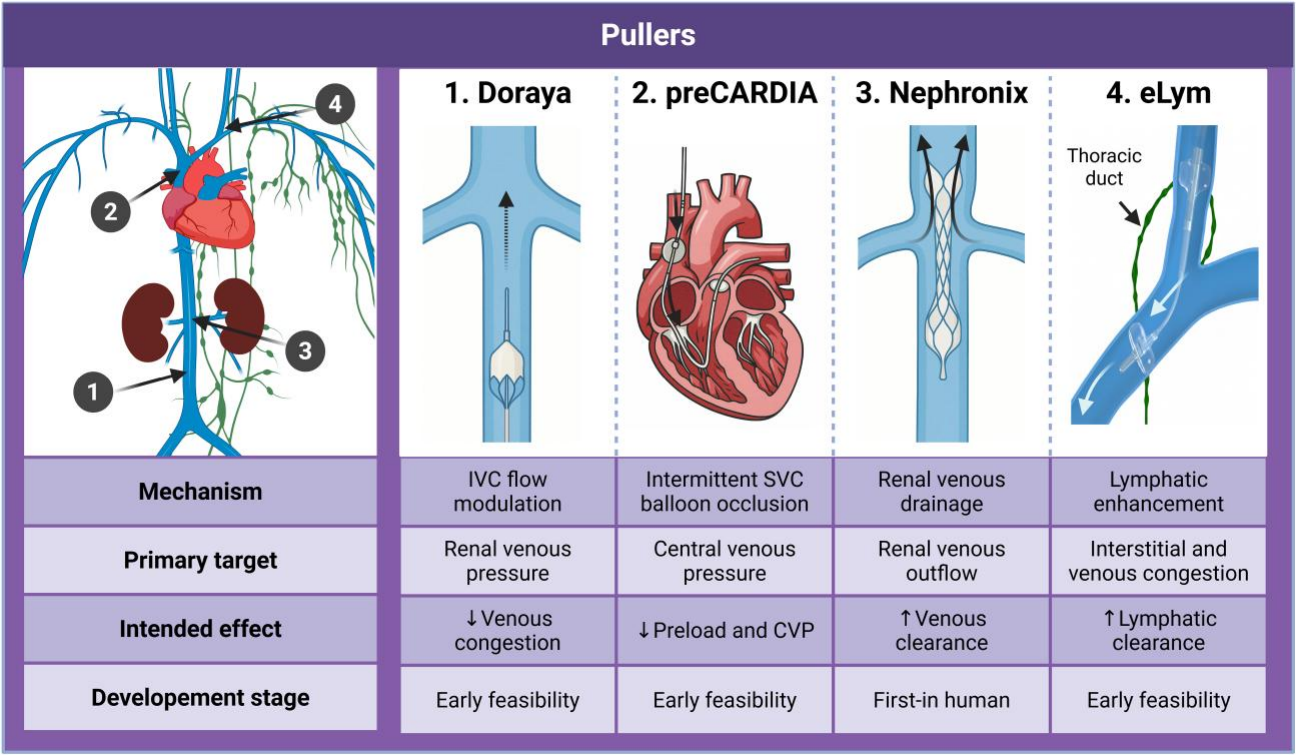
Devices to reduce renal venous pressure (‘pullers’). The figure illustrates the different types of *pullers*. The top left image shows the positioning of each device within the venous–lymphatic system: inferior vena cava (IVC) and superior vena cava (SVC)

In line with the role of venous congestion in the pathophysiology of cardiorenal dysfunction, several device-based strategies have been developed to reduce venous pressures and improve renal outflow conditions (*Figure 1*). These so-called ‘puller’ devices aim to facilitate decongestion by reducing venous and/or lymphatic congestion, thereby potentially improving the transrenal perfusion gradient and improving diuretic responsiveness. Importantly, pullers do not directly remove sodium or fluid, but are intended to act as adjuncts to pharmacological decongestive therapy by targeting venous congestion as a key limiting factor.

### Doraya

The Doraya catheter (Revamp Medical, Netania, Israel) is an intravascular device designed to reduce renal venous congestion by modifying venous pressure conditions within the inferior vena cava. The device is positioned in the inferior vena cava below the level of the renal veins and introduces a controlled, partial narrowing of the caval lumen without causing complete obstruction. This creates a pressure gradient within the inferior vena cava, resulting in lower venous pressure at the level of the renal veins. The first-in-human feasibility study examined the use of the Doraya catheter in nine AHF patients. The procedure appeared safe, as there were no adverse events related to the device.^33,34^ The device created a pressure gradient in the inferior vena cava and resulted in a reduction in renal venous pressures. These haemodynamic changes were associated with increases in diuresis and improvements in congestion markers, although interpretation is limited by the small sample size and lack of a comparator group.^34^ A larger-scale study is currently in progress (NCT05876078).

### PreCardia

The preCARDIA system (Abiomed, Danvers, MA, USA) targets venous congestion through intermittent modulation of central venous return. The device operates via cyclical balloon occlusion of the superior vena cava, consisting of repeated periods of partial occlusion (typically several minutes) followed by brief balloon deflation, allowing controlled and reversible reductions in central venous and cardiac filling pressures. The superior vena cava accounts for approximately 30% of the venous return and its occlusion was shown to result in a modest reduction in filling pressures without a significant effect on blood pressure or cardiac output in a first-in-human study.^35^ Initial feasibility of this approach has been evaluated in 30 ADHF patients in the single arm VENUS-HF early feasibility study, where observations included reductions in right atrial pressure and pulmonary capillary wedge pressure, accompanied by increases in urine output, without evidence of haemodynamic instability.^36^ An expanded report, adding another 30 patients with a second-generation device to the same early feasibility study, has subsequently showed consistent haemodynamic effects in a larger cohort.^37^ Current evidence remains mechanistic in nature, and whether these physiological effects translate into meaningful clinical benefit remains unknown.

### Nephronyx perfusor

The Nephronyx perfusor (Nephronyx Ltd., Modiin, Israel) is a fully intravascular, self-expanding device designed to reduce renal venous congestion by facilitating passive renal venous outflow. The device is deployed percutaneously in the inferior vena cava at the level of the renal veins and remains entirely within the vasculature, without the need for external connections or active energy delivery. Its mechanism of action relies on local modification of caval blood flow. By creating a segment of accelerated flow within the inferior vena cava, the device is intended to generate a localized reduction in intraluminal pressure adjacent to the renal vein ostia. This pressure gradient may promote passive drainage from the renal veins into the cava, thereby lowering renal venous pressure and reducing renal venous afterload. Importantly, the Nephronyx system does not actively pump blood and does not involve extracorporeal circulation. The device is retrievable and can be removed percutaneously using standard snaring techniques. Clinical experience with this approach remains confined to early feasibility and proof-of-concept evaluation, and further studies are required to better define its physiological effects, safety profile, and potential clinical role.

### WhiteSwell eLym™ system

The WhiteSwell eLym™ System (WhiteSwell, Ltd., Galway, Ireland) represents a distinct puller strategy by targeting lymphatic rather than purely intravascular congestion. The device is designed to facilitate thoracic duct drainage into the venous circulation, thereby enhancing lymphatic outflow despite an elevated central venous pressure.^38^ The eLym™ System is a catheter-based system placed near the thoracic duct outflow at the bifurcation of the left internal jugular and subclavian veins. Using controlled local pressure modulation at the thoracic duct outflow, the device aims to facilitate lymphatic drainage into the venous circulation and reduce interstitial congestion. Importantly, this approach is intended to support lymphatic drainage and reduce interstitial congestion, but does not replace pharmacological decongestive therapy aimed at sodium removal. Preclinical studies demonstrated proof of concept for direct interstitial decongestion.^39^ More recently, data from the ongoing prospective, multicentre, single-arm Decongestion of Excess Lymphatic Fluid via the Thoracic Duct in Acute Decompensated Heart Failure (DELTA-HF) study further supported the feasibility of thoracic duct decompression in patients with ADHF and fluid overload.^40^ However, current evidence remains limited to early-phase, non-randomized studies relying predominantly on surrogate congestion markers, while procedural complexity, anatomical constraints, and safety considerations may limit broader applicability. Additionally, while the device aims to improve extravascular congestion, it does so by moving the fluid intravascularly. As such, proper patient selection will be critical as patients with very high filling pressures could theoretically worsen.

Puller devices are associated with potential risks inherent to invasive catheter-based therapies. These include complications related to vascular access, such as local bleeding or vascular trauma, as well as risks associated with device positioning and manipulation. In addition, current puller-based approaches are limited by short treatment duration and typically require systemic anticoagulation, which may increase the risk of bleeding events in this fragile patient population.

## Pushers

**Figure 2:**
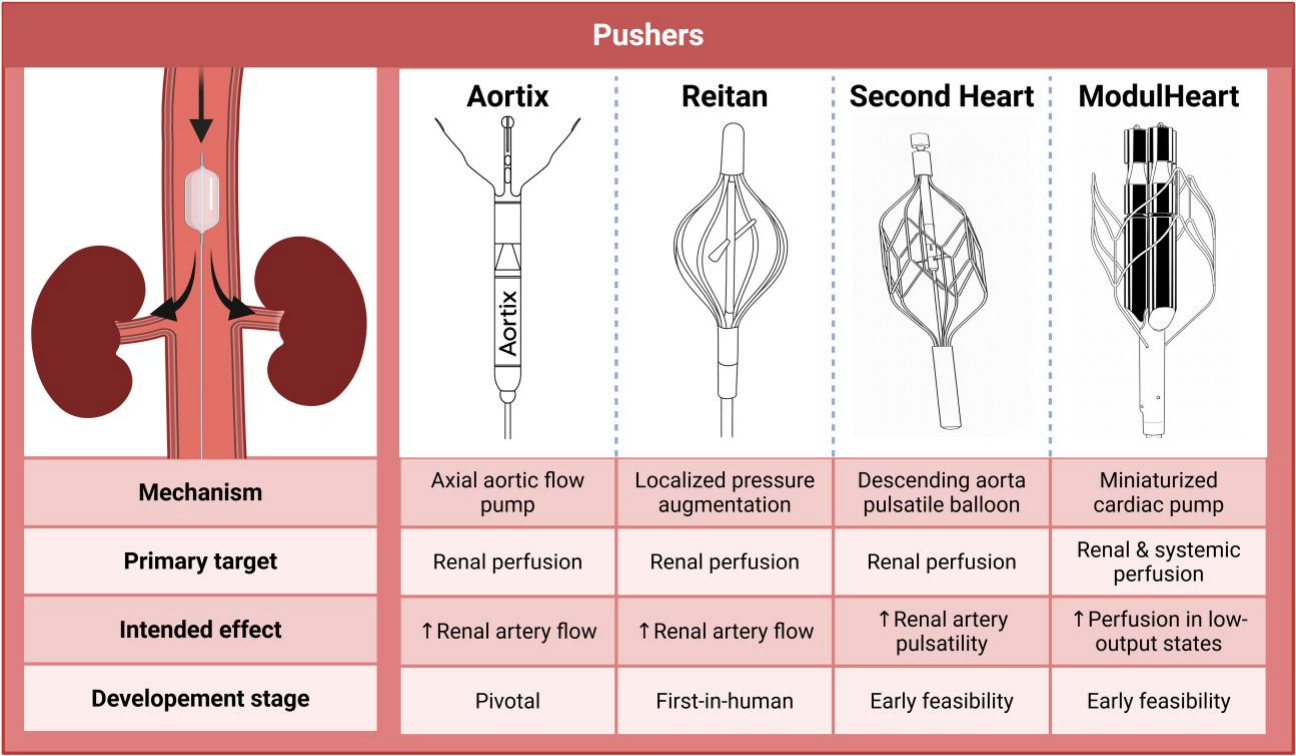
Devices to enhance renal perfusion (‘pushers’). The figure illustrates the different types of *pushers*. All these devices are positioned in the suprarenal aorta and increase renal perfusion. The top left image depicts the general mechanism of action of these devices

In contrast to puller-based approaches that primarily target venous and lymphatic congestion, pusher devices are designed to augment forward flow and arterial perfusion in patients with HF (*Figure 2*). The underlying rationale is that improving renal haemodynamics may facilitate decongestion in selected patients.

### Aortix

The Aortix system (Procyrion, Houston, TX, USA) is a micro-axial pump temporarily implanted in the descending aorta above the renal arteries. The device augments forward flow by accelerating blood flow through the aorta and its branches, including the renal arteries, with the aim of improving arterial perfusion and renal blood flow. Preclinical studies showed reductions in left ventricular pre- and afterload and systemic vascular resistance, accompanied by increases in renal artery flow of up to 36% and renal perfusion pressure of up to 75%.^41,42^ An early feasibility study in patients with ADHF, persistent congestion and WRF reported improvements in both left- and right-sided filling pressures.^43^ Device support was associated with increased urine output, net fluid loss and measures of diuretic response. In the single-arm study, a sustained trend towards improvement lasting up to 30 days after device removal was observed following use of the device, together with reductions in dyspnoea and NT-proBNP levels, and decreases in loop diuretic doses. Most adverse events were related to vascular access, including bleeding or vascular injury, some requiring endovascular or surgical intervention, with no reports of pump malfunction or thrombosis. Patients with hypotension or requiring high-dose inotropic support were excluded from these early studies.

Following these pilot observations, the randomized DRAIN-HF trial (NCT05677100) is currently underway to evaluate safety, decongestive efficacy, and 30-day outcomes in a similar patient population with an insufficient response to high-dose intravenous loop diuretics.

### Reitan

The Reitan catheter pump (CardioBridge, Hechingen, Germany) employs a rotating propeller enclosed within a cage to generate a pressure gradient and augment aortic blood flow. The device is implanted in the descending aorta below the left subclavian artery and provides greater circulatory support than an intra-aortic balloon pump, while remaining less invasive than axial-flow ventricular assist devices.

The system was initially evaluated in a first-in-man safety and feasibility study in patients undergoing high-risk percutaneous coronary intervention, showing effective haemodynamic support with minimal haemolysis.^44^ Subsequently, the Reitan catheter pump was studied in patients admitted with ADHF, low cardiac index and significant renal dysfunction requiring inotropic or mechanical support.^45^ In this prospective multicentre experience, device support was associated with a significant increase in cardiac index, accompanied by reductions in central venous pressure and improvements in urine output. These haemodynamic changes were paralleled by a reduction in serum creatinine and an increase in estimated glomerular filtration rate, despite no net increase in diuretic or inotropic therapy. More recently, a first-in-man case report described use of the 10F next-generation Reitan catheter pump in a patient with severe cardiorenal syndrome, reporting favourable haemodynamic effects and increasing urine output during short-term support.^46^

With respect to safety, higher pump speeds were associated with mild haemolysis, reflected by transient increases in plasma free haemoglobin that remained below the upper reference limit, together with a small post-procedural decrease in haemoglobin concentration.

### Second heart assist

The Second Heart Assist (Second Heart Assist Inc., Salt Lake City, UT, USA) system is a catheter-based, propeller-driven mechanical circulatory support device housed within a stent cage and deployed in the descending aorta above the renal arteries. By augmenting pulsatile aortic flow, the device is intended to increase arterial perfusion, including renal arterial flow, while reducing left ventricular afterload.

Early clinical and mechanistic studies suggest generation of up to approximately 2.5 l/min of additional pulsatile flow, with associated increases in renal artery flow of up to 50%.^47^ A distinctive feature of the second-generation Second Heart Assist Freedom system is its wirelessly powered, driveline-free design, potentially allowing ambulatory support.

Current evidence remains limited to first-in-man and early clinical experience, without randomized data or demonstrated benefit on hard clinical outcomes.

### ModulHeart

The ModulHeart system (Puzzle Medical Devices, Montreal, Canada) consists of three small axial-flow pumping elements positioned in parallel within the descending aorta. Forward flow is augmented by distributing the pumping work across multiple low-speed axial pumps. This design intends to increase in aortic flow and perfusion pressure while limiting shear stress and potentially reducing the risk of haemolysis.

The system has been evaluated as a cardiorenal mechanical circulatory support device during high-risk percutaneous coronary intervention in a first-in-man clinical experience including four male patients. Device support was associated with reductions in central venous pressure, pulmonary artery pressure and left ventricular end-diastolic pressure, together with an increase in cardiac index.^48^ In addition, urine output increased within 15 min after implantation, while serum creatinine remained stable over the subsequent 24 h. No signs of haemolysis were reported during device support. An early feasibility study evaluating the ModulHeart system in patients with ADHF and diuretic resistance is currently planned (NCT06174623).

Pusher-based devices share the objective of augmenting arterial perfusion but differ substantially in pumping principles, invasiveness, and intended duration of support. Although several devices have been associated with favourable haemodynamic changes, effects on natriuresis and durable decongestion remain uncertain. In addition, several of these technologies function primarily as temporary circulatory support systems rather than renal-targeted therapies.

The available clinical evidence remains limited to small, single-arm, early feasibility studies demonstrating haemodynamic and short-term physiological effects without robust outcome data. Furthermore, these devices require large-bore arterial access and systemic anticoagulation, which may limit broader applicability. From a cardiorenal perspective, pusher-based strategies may be most relevant in selected patients with true low-output physiology, but they should currently be regarded as investigational adjuncts rather than established therapies.

## Removers

**Figure 3:**
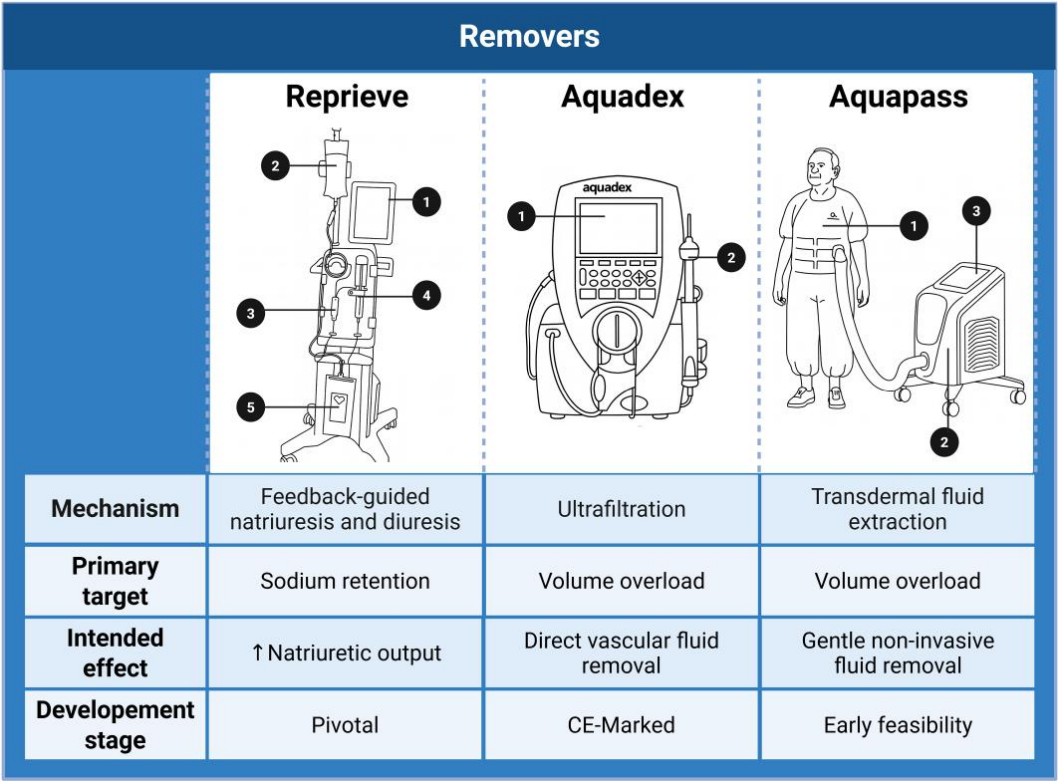
Devices to enhance fluid removal (‘removers’). The figure illustrates the different types of removers. The numbers in each panel indicate the most relevant parts of each device. Reprieve: touchscreen console (1), saline bag (2), IV saline bag infuser (3), furosemide IV infuser (4), urine collection bag with flow sensor (5); Aquadex: display screen (1), hemofilter (2); Aquapass: wearable microclimate garment (1), microclimate generator (2), control unit (3)

In contrast to strategies targeting haemodynamics, ‘remover’ devices aim to directly reduce congestion through active sodium and/or fluid removal (*Figure 3*). These approaches either enhance diuresis under controlled conditions or provide alternative routes for sodium and fluid removal beyond the kidneys, including extracorporeal or transcutaneous strategies.

### Reprieve system

The Reprieve System® (Reprieve Cardiovascular, Milford, MA, USA) is a semi-automated diuretic infusion platform designed to individualize sodium and fluid removal during ADHF. The system integrates a bedside console with a Foley catheter and intravenous access, enabling real-time monitoring of urine output and IV loop diuretic administration. Based on urine volume and calculated natriuresis, the system semi-automatically adjusts loop diuretic dosing and concomitant isotonic saline infusion to achieve a predefined net fluid balance while avoiding excessive intravascular volume depletion. By coupling loop diuretic administration to urinary sodium excretion, Reprieve aims to optimize natriuresis rather than fluid removal alone.

In early feasibility studies (TARGET-1 and TARGET-2), use of the Reprieve system was associated with substantially higher urine output and effective sodium removal compared with preceding conventional diuretic therapy, alongside stable blood pressure and preservation of renal function.^49^ A subsequent early feasibility study in patients hospitalized with ADHF similarly demonstrated precise and controllable volume removal without clear evidence of impaired renal perfusion.^50^ More recently, the randomized pilot Fluid management of Acute decompensated heart failure Subjects Treated with Reprieve System (FASTR) trial evaluated the Reprieve system against optimal diuretic therapy in patients hospitalized for ADHF.^51^ The use of the Reprieve system resulted in faster decongestion and shorter duration of intravenous diuretic treatment without an increase in safety events. However, the study remained limited by its pilot design and reliance on physiological and decongestive endpoints rather than hard clinical outcomes. The FASTRII trial is currently ongoing (NCT06898515), which incorporates some harder clinical end points, but again will largely rely on physiologic endpoints given its modest sample size.

### Aquadex®: extracorporeal ultrafiltration

Extracorporeal ultrafiltration allows for the direct removal of isotonic fluid independent of renal function. The Aquadex SmartFlow® system (Nuwellis, Eden Prairie, MN, USA) is a portable ultrafiltration platform developed specifically for HF patients. It allows controlled removal of plasma water through a venovenous extracorporeal circuit using a filtration membrane, typically through a small central venous catheter. Ultrafiltration rates are generally kept low (approximately 100–250 ml/h) to achieve gradual net fluid removal with the goal of reducing the risk of intravascular depletion.

Clinical experience with ultrafiltration in ADHF has included several randomized and non-randomized studies, with mixed findings. In the UNLOAD trial, which enrolled 200 patients, ultrafiltration was associated with greater weight loss at 48 h (5.0 vs 3.1 kg) and fewer HF rehospitalizations at 90 days compared with intravenous diuretics.^52^ In contrast, the CARRESS-HF trial, which randomized 188 patients with cardiorenal syndrome compared a stepped pharmacologic diuretic strategy with ultrafiltration using the Aquadex system and found no superiority of ultrafiltration with respect to weight loss, while renal function worsened and adverse events, including hypotension and bleeding, were more frequent in the ultrafiltration group.^53^ Subsequently, the AVOID-HF trial enrolled 224 patients and found a longer time to first HF event with ultrafiltration, but was terminated prematurely and therefore remained inconclusive.^54^

In line with these findings, current ESC HF guidelines recommend that ultrafiltration should be considered in selected patients with persistent congestion refractory to optimized medical therapy.^55^ To date, ultrafiltration remains the device-based decongestive strategy supported by the largest body of clinical trial data and likely has a more potent direct decongestive effect than the majority of therapies described above. However, its notable absence in routine clinical care of AHF patients underscores the challenges these devices will face.

### Aquapass®: sweat-inducing therapy

Aquapass® (AquaPass Medical Ltd., Israel) is a non-invasive, wearable device designed to promote fluid and sodium removal by enhancing sweat production. The system consists of externally applied heating elements that increase local skin temperature for several hours, thereby stimulating eccrine sweat gland activity and inducing controlled perspiration.

Clinical evaluation of Aquapass® has so far been limited to early feasibility studies in hospitalized patients with HF. These small, non-randomized studies demonstrated that controlled sweat induction is feasible without major haemodynamic or renal instability.^56,57^ Experience has also been extended to short-term outpatient use following hospital discharge, suggesting technical feasibility beyond the in-hospital setting.^57^

The non-invasive nature of Aquapass® distinguishes it from other device strategies and avoids risks related to vascular access or extracorporeal circulation. In addition, current evidence remains confined to small feasibility studies, and the contribution of sweat-induced fluid removal to sustained decongestion, as well as long-term adherence and skin tolerance, requires further evaluation.

### Direct sodium removal

Direct sodium removal (DSR) is a therapeutic concept specifically designed to promote sodium removal through intermittent infusion of a sodium-free solution into the peritoneal cavity, allowing sodium diffusion followed by drainage. Clinical experience with this approach has primarily been obtained using the Alfapump DSR system (Sequana Medical, Belgium), an implantable subcutaneous pump connected to a peritoneal catheter and adapted from its original indication in refractory ascites.

In two exploratory, single-arm studies, DSR therapy was associated with near normalization of diuretic responsiveness, lasting for several months, in patients with advanced HF and severe diuretic resistance^58^. However, catheter failure (occlusion) was extremely frequent, and clinical development of the Alfapump system for the HF indication has been discontinued.

### Non-device thermal fluid removal strategies

Although sauna therapy is not a device-based intervention, controlled thermal exposure using standardized infrared systems may be considered as part of non-pharmacological approaches to fluid removal.^59^ In patients with HF, small studies and meta-analyses have reported improvements in symptoms, exercise capacity, and surrogate markers such as natriuretic peptide levels^60–63^ However, these data are limited and heterogeneous, and their relevance for decongestion in cardiorenal syndrome remains uncertain. Importantly, systemic vasodilation and heat exposure may raise safety concerns in vulnerable HF patients. Therefore, sauna therapy should be considered separately from device-based decongestive strategies.

Remover strategies differ substantially in their mechanisms of decongestion. While some approaches aim to optimize diuretic-driven natriuresis, others rely on direct extracorporeal fluid removal, peritoneal sodium extraction, or sweat-mediated fluid loss. Currently, extracorporeal ultrafiltration remains the only remover strategy supported by randomized data and incorporated into clinical guidelines, albeit with a restricted indication in selected patients with refractory congestion. In contrast, the other remover-based technologies are only supported by early feasibility studies and physiological endpoints. In the absence of robust clinical outcome data, their role in routine clinical practice remains unclear.

## Framework for devices in cardiorenal syndrome

Interpretation of device trials in cardiorenal dysfunction has been challenging because of heterogeneity in patient selection and study design. The multiplicity of devices and therapeutic strategies reflects the limited evidence on the optimal decongestion approach to improve clinical outcomes. The optimal reference diuretic treatment remains to be clearly defined and standardized across clinical settings (ADHF, cardiogenic shock, advanced HF, etc.). Many trials enrol patients under a broad cardiorenal label that encompasses diverse clinical presentations and pathophysiological states. Changes in kidney function during decongestive therapy are frequently used both to define cardiorenal involvement and to assess treatment response, even though such changes are highly dynamic, context-dependent, and not necessarily indicative of clinically relevant renal injury.^3^

Appropriate patient selection based on the intended physiological target of the device remains essential. In practice, venous congestion, altered sodium handling, and changes in renal perfusion rarely occur in isolation, but represent interrelated manifestations of the decompensated HF state. Device evaluation should acknowledge this overlap rather than rely on rigid phenotypic categorization. In addition, background medical therapy prior to inclusion (e.g. intravenous diuretic dosing, combination strategies, infusion protocols, and vasoactive treatments) is often insufficiently standardized. Similarly, there is no consensus on how to define key inclusion criteria such as diuretic resistance, congestion, or low cardiac output, further contributing to heterogeneity across studies.

For devices applied during acute decompensation and intended for short-term use, the initial objective should be to demonstrate a reproducible physiological effect under controlled conditions before proceeding to larger outcome studies. Early-phase investigations should therefore minimize confounding by concomitant intravenous therapies where feasible and focus on direct haemodynamic effects, natriuretic response, urine output, renal function, and objective measures of congestion. This is particularly relevant as recent studies evaluating intensified pharmacological decongestion strategies have demonstrated improvements in natriuresis and diuresis without consistent effects on hard clinical outcomes.^64,65^ Such studies establish whether the proposed mechanism of action results in measurable decongestion or haemodynamic improvement.

Following demonstration of reproducible physiological efficacy, a randomized pilot study should evaluate the device against contemporary medical care before proceeding to larger outcome trials. Subsequent pivotal randomized studies should evaluate device therapy on top of optimized contemporary decongestive care versus optimized care alone, preferably using sham-controlled designs when technically and ethically feasible. Importantly, the comparator arm should reflect current HF management standards, as optimization of pharmacological decongestion and guideline-directed therapy alone may already substantially improve decongestion and clinical outcomes^64–68^ Device-based interventions should therefore demonstrate benefit beyond what can be achieved with structured contemporary medical care. Importantly, improvements in natriuresis, decongestion, haemodynamics, or other surrogate endpoints should not be assumed to translate into improved long-term clinical outcomes. Given their invasiveness, procedural risks, and costs, meaningful clinical benefit will need to be demonstrated before adoption in routine clinical practice.

Most current technologies have been developed for temporary use during ADHF, which remains the most appropriate setting for initial evaluation. However, the role of repeated or longer-term device therapy remains largely unexplored.

The typical objectives and evidentiary expectations across different stages of device development are summarized in *Table 1*.

**Table 1:** Typical objectives and endpoints across stages of device development in cardiorenal syndrome

Development stage	Primary objective	Typical endpoints
**Preclinical/*in vitro***	Technical feasibility and mechanistic validation	Flow dynamics, pressure changes, device performance, biocompatibility
**First-in-human**	Initial safety and proof of concept in humans	Procedural success, acute haemodynamic effects, immediate safety signals
**Early feasibility**	Reproducibility of physiological efficacy and decongestive effects	Changes in congestion markers, diuretic response, renal parameters, symptoms
**Pilot trial**	Preliminary evaluation of clinical benefit versus optimized contemporary care	Changes in congestion markers, diuretic response renal parameters, symptoms, exploratory clinical outcomes
**Pivotal trial**	Evaluation of incremental clinical benefit and safety versus optimized contemporary care	HF hospitalization, mortality, quality of life, safety endpoints
**Post-marketing**	Long-term safety, effectiveness, and patient selection	Rare adverse events, durability of effect, real-world utilization

## Limitations of device-based therapies in cardiorenal syndrome

Beyond the device-specific considerations discussed above, several major limitations continue to restrict the clinical integration of device-based therapies in cardiorenal syndrome (*Table 2*). Most available evidence comes from small, early-phase or proof-of-concept studies focused on haemodynamic or surrogate physiological endpoints, generally without a control group or outcome data. The risk of bias is substantial, particularly because many studies lack any form of controls, include highly selected patients, and compare device-based interventions with control strategies that may differ in monitoring intensity, background decongestive therapy, or frequency of clinical follow-up.

**Table 2:** Major limitations and challenges of device-based therapies in cardiorenal syndrome

Evidence-based limitations
Predominance of small, early-phase or proof-of-concept studies; limited randomized evidence
Reliance on surrogate physiological markers (e.g. central hemodynamics) rather than clinical outcomes
Predominantly observational data with limited internal validity
Lack of sham-controlled designs, increasing susceptibility to bias
Limited evidence of sustained clinical benefit beyond physiological effects

Endpoint and definition heterogeneity
Inconsistent reporting of efficacy metrics (e.g. urine output, natriuretic response, biomarkers)
Heterogeneous and non-standardized endpoints
Lack of standardized definitions (e.g. diuretic resistance)
Limited ability to perform reliable cross-device comparisons

Patient selection and generalizability
Invasive profile limiting applicability to selected patients
Unclear identification of optimal responder populations
Dependence on haemodynamic profile and congestion phenotype
Likely most relevant in advanced HF populations
Limited scalability

Procedural and organizational requirements
Need for specialized clinical settings (e.g. advanced HF units, ICU-level care)
Requirement for trained personnel
Need for strict intraprocedural monitoring
Institutional expertise and infrastructure requirements

Safety and risk considerations
Procedural risks and device-related complications
Adverse events requiring careful monitoring

Economic and health-system impact
Direct device-related costs
Implementation and training requirements
Increased personnel and resource utilization
Broader health-economic implications
Potential limitations in real-world availability

Study heterogeneity further limits interpretation. Patient selection, definitions of diuretic resistance, congestion, haemodynamic profiles, and treatment response vary widely across studies, making cross-device comparisons difficult. In addition, treatment response may depend on whether the device mechanism matches the dominant pathophysiological driver in an individual patient. Venous congestion, impaired perfusion, and altered sodium handling often coexist and evolve during decompensation and treatment, which may partly explain variable effects across studies. Finally, the invasive nature, procedural complexity, and resource requirements of the majority of these devices restrict their applicability to selected patients and specialized centres.

## Conclusion

Congestion remains a key determinant of symptoms, hospitalization, and organ dysfunction in HF. In most patients, decongestion can be achieved with pharmacological therapy when applied within a structured and optimized treatment strategy. Device-based approaches have been developed as adjuncts to medical therapy, when congestion persists despite appropriate care.

These devices target different, often overlapping components of cardiorenal pathophysiology, including venous congestion, altered renal perfusion, lymphatic dysfunction, and impaired renal sodium handling. Their mechanisms, invasiveness, and clinical maturity vary widely, and it remains unclear which patients could benefit from these devices. At present, most devices remain in an early stage of development, with predominantly mechanistic and physiological signals rather than proven clinical benefit. Extracorporeal ultrafiltration remains the most extensively studied device-based strategy and the only one incorporated into clinical guidelines, with a restricted indication in selected patients with refractory congestion.

Future studies should first demonstrate reproducible physiological efficacy, followed by randomized evaluation against optimized contemporary decongestive therapies. Larger, methodologically robust trials will be required to determine whether these devices improve clinical outcomes beyond existing pharmacological strategies.

## Declarations

### Data Availability

No data were generated or analysed for this manuscript.

## References

[1] Damman K, Valente MAE, Voors AA, O’Connor CM, Van Veldhuisen DJ, Hillege HL. Renal impairment, worsening renal function, and outcome in patients with heart failure: an updated meta-analysis. Eur Heart J 2014;35:455–69. 10.1093/eurheartj/eht386 24164864

[2] House AA, Wanner C, Sarnak MJ, Piña IL, McIntyre CW, Komenda P, et al Heart failure in chronic kidney disease: conclusions from a kidney disease: improving global outcomes (KDIGO) controversies conference. Kidney Int 2019;95:1304–17. 10.1016/j.kint.2019.02.022 31053387

[3] Ahmad T, Jackson K, Rao VS, Tang WHW, Brisco-Bacik MA, Chen HH, et al Worsening renal function in patients with acute heart failure undergoing aggressive diuresis is not associated with tubular injury. Circulation 2018;137:2016–28. 10.1161/CIRCULATIONAHA.117.030112 29352071 PMC6066176

[4] Mullens W, Damman K, Testani JM, Martens P, Mueller C, Lassus J, et al Evaluation of kidney function throughout the heart failure trajectory—a position statement from the Heart Failure Association of the European Society of Cardiology. Eur J Heart Fail 2020;22:584–603. 10.1002/ejhf.1697 31908120

[5] Crespo-Leiro MG, Metra M, Lund LH, Milicic D, Costanzo MR, Filippatos G, et al Advanced heart failure: a position statement of the Heart Failure Association of the European Society of Cardiology. Eur J Heart Fail 2018;20:1505–35. 10.1002/ejhf.1236 29806100

[6] Chonko AM, Bay WH, Stein JH, Ferris TF. The role of renin and aldosterone in the salt retention of edema. Am J Med 1977;63:881–9. 10.1016/0002-9343(77)90541-1 605909

[7] Patel KP, Katsurada K, Zheng H. Cardiorenal syndrome: the role of neural connections between the heart and the kidneys. Circ Res 2022;130:1602–1617. 10.1161/CIRCRESAHA.122.319989 PMC9179008 35549375

[8] Ljungman S, Laragh JH, Cody RJ. Role of the kidney in congestive heart failure. Relationship of cardiac index to kidney function. Drugs 1990;39:10–21. 10.2165/00003495-199000394-00004 2354670

[9] Guzik M, Sokolski M, Hurkacz M, Zdanowicz A, Iwanek G, Marciniak D, et al Serum osmolarity and vasopressin concentration in acute heart failure-influence on clinical course and outcome. Biomedicines 2022;10:2034. 10.3390/biomedicines10082034 36009581 PMC9405797

[10] Biegus J, Nawrocka-Millward S, Zymliński R, Fudim M, Testani J, Marciniak D, et al Distinct renin/aldosterone activity profiles correlate with renal function, natriuretic response, decongestive ability and prognosis in acute heart failure. Int J Cardiol 2021;345:54–60. 10.1016/j.ijcard.2021.10.149 34728260

[11] Zymliński R, Sierpiński R, Metra M, Cotter G, Sokolski M, Siwołowski P, et al Elevated plasma endothelin-1 is related to low natriuresis, clinical signs of congestion, and poor outcome in acute heart failure. ESC Heart Fail 2020;7:3536–44. 10.1002/ehf2.13064 33063475 PMC7755016

[12] Fallick C, Sobotka PA, Dunlap ME. Sympathetically mediated changes in capacitance redistribution of the venous reservoir as a cause of decompensation. Circ Heart Fail 2011;4:669–75. 10.1161/CIRCHEARTFAILURE.111.961789 21934091

[13] Miller WL . Fluid volume overload and congestion in heart failure. Circ Heart Fail 2016;9:e002922. 10.1161/CIRCHEARTFAILURE.115.002922 27436837

[14] Mullens W, Abrahams Z, Francis GS, Sokos G, Taylor DO, Starling RC, et al Importance of venous congestion for worsening of renal function in advanced decompensated heart failure. J Am Coll Cardiol 2009;53:589–96. 10.1016/j.jacc.2008.05.068 19215833 PMC2856960

[15] Bobbio E, Bollano E, Polte CL, Ekelund J, Rådegran G, Lundgren J, et al Association between central haemodynamics and renal function in advanced heart failure: a nationwide study from Sweden. ESC Heart Fail 2022;9:2654–63. 10.1002/ehf2.13990 35611889 PMC9288757

[16] Boorsma EM, terMaaten JM, Voors AA, van Veldhuisen DJ. Renal compression in heart failure: the renal tamponade hypothesis. JACC Heart Fail 2022;10:175–83. 10.1016/j.jchf.2021.12.005 35241245

[17] Biegus J, Zymliński R, Testani J, Marciniak D, Zdanowicz A, Jankowska EA, et al Renal profiling based on estimated glomerular filtration rate and spot urine sodium identifies high-risk acute heart failure patients. Eur J Heart Fail 2021;23:729–39. 10.1002/ejhf.2053 33190378

[18] Łagosz P, Sokolski M, Biegus J, Tycinska A, Zymlinski R. Elevated intra-abdominal pressure: a review of current knowledge. World J Clin Cases 2022;10:3005–13. 10.12998/wjcc.v10.i10.3005 35647129 PMC9082714

[19] Mullens W, Abrahams Z, Skouri HN, Francis GS, Taylor DO, Starling RC, et al Elevated intra-abdominal pressure in acute decompensated heart failure: a potential contributor to worsening renal function? J Am Coll Cardiol 2008;51:300–6. 10.1016/j.jacc.2007.09.043 18206740

[20] Łagosz P, Biegus J, Lewandowski Ł, Ponikowski P, Zymliński R. Prevalence of increased intra-abdominal pressure and its impact on renal function in acute decompensated heart failure: a prospective pilot study. Kardiol Pol 2024;82:292–302. 10.33963/v.phj.99497 38493453

[21] Salah HM, Biegus J, Fudim M. Role of the renal lymphatic system in heart failure. Curr Heart Fail Rep 2023;20:113–20. 10.1007/s11897-023-00595-0 36848025

[22] Ponikowska B, Fudim M, Iwanek G, Zymliński R, Biegus J. Harnessing the lymphatic system. Heart Fail Rev 2025;30:673–83. 10.1007/s10741-024-10449-z 39402329 PMC12165986

[23] Witte MH, Dumont AE, Clauss RH, Rader B, Levine N, Breed ES. Lymph circulation in congestive heart failure. Circulation 1969;39:723–33. 10.1161/01.CIR.39.6.723 5785287

[24] Dumont AE, Clauss RH, Reed GE, Tice DA. Lymph drainage in patients with congestive heart failure. N Engl J Med 1963;269:949–52. 10.1056/NEJM196310312691804 14056641

[25] Fudim M, Salah HM, Sathananthan J, Bernier M, Pabon-Ramos W, Schwartz RS, et al Lymphatic dysregulation in patients with heart failure: JACC review topic of the week. J Am Coll Cardiol 2021;78:66–76. 10.1016/j.jacc.2021.04.090 34210416

[26] Hanberg JS, Sury K, Wilson FP, Brisco MA, Ahmad T, Ter Maaten JM, et al Reduced cardiac index is not the dominant driver of renal dysfunction in heart failure. J Am Coll Cardiol 2016;67:2199–208. 10.1016/j.jacc.2016.02.058 27173030 PMC4867078

[27] Chioncel O, Mebazaa A, Harjola V-P, Coats AJ, Piepoli MF, Crespo-Leiro MG, et al Clinical phenotypes and outcome of patients hospitalized for acute heart failure: the ESC heart failure long-term registry. Eur J Heart Fail 2017;19:1242–54. 10.1002/ejhf.890 28463462

[28] Mullens W, Verbrugge FH, Nijst P, Tang WHW. Renal sodium avidity in heart failure: from pathophysiology to treatment strategies. Eur Heart J 2017;38:1872–82. 10.1093/eurheartj/ehx035 28329085

[29] Mullens W, Damman K, Harjola V-P, Mebazaa A, Brunner-La Rocca H-P, Martens P, et al The use of diuretics in heart failure with congestion—a position statement from the Heart Failure Association of the European Society of Cardiology. Eur J Heart Fail 2019;21:137–55. 10.1002/ejhf.1369 30600580

[30] Damman K, Testani JM. The kidney in heart failure: an update. Eur Heart J 2015;36:1437–44. 10.1093/eurheartj/ehv010 25838436 PMC4465636

[31] Cox ZL, Rao VS, Ivey-Miranda JB, Moreno-Villagomez J, Mahoney D, Ponikowski P, et al Compensatory post-diuretic renal sodium reabsorption is not a dominant mechanism of diuretic resistance in acute heart failure. Eur Heart J 2021;42:4468–77. 10.1093/eurheartj/ehab620 34529781 PMC8599022

[32] Biegus J, Zymliński R, Testani J, Fudim M, Cox ZL, Guzik M, et al The blunted loop diuretic response in acute heart failure is driven by reduced tubular responsiveness rather than insufficient tubular delivery. The role of furosemide urine excretion on diuretic and natriuretic response in acute heart failure. Eur J Heart Fail 2023;25:1323–33. 10.1002/ejhf.2852 37042083

[33] Zymliński R, Dierckx R, Biegus J, Vanderheyden M, Bartunek J, Ponikowski P. Novel IVC doraya catheter provides congestion relief in patients with acute heart failure. JACC Basic Transl Sci 2022;7:326–7. 10.1016/j.jacbts.2022.02.013 35411326 PMC8993904

[34] Zymliński R, Biegus J, Vanderheyden M, Gajewski P, Dierckx R, Bartunek J, et al Safety, feasibility of controllable decrease of vena cava pressure by doraya catheter in heart failure. JACC Basic Transl Sci 2023;8:394–402. 10.1016/j.jacbts.2023.02.010 37138800 PMC10149648

[35] Kapur NK, Karas RH, Newman S, Jorde L, Chabrashvili T, Annamalai S, et al First-in-human experience with occlusion of the superior vena cava to reduce cardiac filling pressures in congestive heart failure. Catheter Cardiovasc Interv 2019;93:1205–10. 10.1002/ccd.28326 31112633 PMC6814201

[36] Kapur NK, Kiernan MS, Gorgoshvili I, Yousefzai R, Vorovich EE, Tedford RJ, et al Intermittent occlusion of the superior vena cava to improve hemodynamics in patients with acutely decompensated heart failure: the VENUS-HF early feasibility study. Circ Heart Fail 2022;15:E008934. 10.1161/CIRCHEARTFAILURE.121.008934 35000420

[37] Yousefzai R, Bhimaraj A, Kiernan MS, Abraham J, Gorgoshvili I, Tedford RJ, et al Mechanically reducing cardiac preload with the preCARDIA system in acutely decompensated heart failure. Heart Fail 2025;14:102841. 10.1016/j.jchf.2025.102841 41389079

[38] Biegus J, Lindenfeld J, Felker G, Bakris G, Jonas M, Lala A, et al Design and rationale of the eLym^TM^ system for decompensation of excess lymphatic fluid via the thoracic duct in acute heart failure (DELTA-HF). ESC Heart Fail 2025;12:1719–26. 10.1002/ehf2.15192 39716986 PMC12055369

[39] Abraham WT, Jonas M, Dongaonkar RM, Geist B, Ueyama Y, Render K, et al Direct interstitial decongestion in an animal model of acute-on-chronic ischemic heart failure. JACC Basic Transl Sci 2021;6:872–81. 10.1016/j.jacbts.2021.09.008 34869951 PMC8617571

[40] Nuche J, García-Cosío Carmena MD, Biegus J, Kereselidze Z, Chukhrukidze A, Khabeishvili G, et al Safety and feasibility of the eLymTM system for interstitial decongestion in acute decompensated heart failure: primary results of the DELTA-HF trial. Eur J Heart Fail 2026:xuag157. 10.1093/ejhf/xuag157 42106950

[41] Annamalai SK, Esposito ML, Reyelt LA, Natov P, Jorde LE, Karas RH, et al Abdominal positioning of the next-generation intra-aortic fluid entrainment pump (Aortix) improves cardiac output in a swine model of heart failure. Circ Heart Fail 2018;11:e005115. 10.1161/CIRCHEARTFAILURE.118.005115 30354560 PMC6203333

[42] Shabari FR, George J, Cuchiara MP, Langsner RJ, Heuring JJ, Cohn WE, et al Improved hemodynamics with a novel miniaturized intra-aortic axial flow pump in a porcine model of acute left ventricular dysfunction. ASAIO Journal 2013;59:240–5. 10.1097/MAT.0b013e31828a6e74 23644610

[43] Cowger JA, Basir MB, Baran DA, Hayward CS, Rangaswami J, Walton A, et al Safety and performance of the aortix device in acute decompensated heart failure and cardiorenal syndrome. JACC Heart Fail 2023;11:1565–75. 10.1016/j.jchf.2023.06.018 37804307

[44] Smith EJ, Reitan O, Keeble T, Dixon K, Rothman MT. A first-in-man study of the reitan catheter pump for circulatory support in patients undergoing high-risk percutaneous coronary intervention. Catheter Cardiovasc Interv 2009;73:859–65. 10.1002/ccd.21865 19455649

[45] Keeble TR, Karamasis GV, Rothman MT, Ricksten S-E, Ferrari M, Hullin R, et al Percutaneous haemodynamic and renal support in patients presenting with decompensated heart failure: a multi-centre efficacy study using the Reitan Catheter Pump (RCP). Int J Cardiol 2019;275:53–8. 10.1016/j.ijcard.2018.09.085 30342873

[46] Napp LC, Mariani S, Ruhparwar A, Schmack B, Keeble TR, Reitan O, et al First-in-man use of the percutaneous 10F reitan catheter pump for cardiorenal syndrome. ASAIO Journal 2022;68:E99–E101. 10.1097/MAT.0000000000001498 35649225

[47] Miller L, Cunningham M, Richardson A, Burton B. Wireless powered and water-proof mechanical circulatory support device for ambulatory class III heart failure. JACC Basic Transl Sci 2022;7:328–9. 10.1016/j.jacbts.2022.03.001 35411316 PMC8993901

[48] Georges G, Trudeau F, Doucet-Martineau J, Rochon M, Potvin J, Ebner A, et al First-in-human experience with the ModulHeart device for mechanical circulatory support and renal perfusion. J Soc Cardiovasc Angiogr Interv 2022;1:100449. 10.1016/j.jscai.2022.100449 39132369 PMC11307613

[49] Biegus J, Zymlinski R, Siwolowski P, Testani J, Szachniewicz J, Tycińska A, et al Controlled decongestion by reprieve therapy in acute heart failure: results of the TARGET-1 and TARGET-2 studies. Eur J Heart Fail 2019;21:1079–87. 10.1002/ejhf.1533 31127666

[50] Ivey-Miranda JB, Rao VS, Shaburishvili T, Verulava I, Khabeishvili N, Petrie MC, et al Reprieve system for the treatment of patients with acute decompensated heart failure. J Card Fail 2025;31:1085–9. 10.1016/j.cardfail.2025.02.002 39947422

[51] Udelson JE, Javaheri A, Fudim M, Damman K, Biegus J, Afzal A, et al Fluid management of acute heart failure with the reprieve system: the randomized controlled FASTR trial. JACC Heart Fail 2026:103062. 10.1016/j.jchf.2026.103062. Epub ahead of print. 42068320

[52] Costanzo MR, Guglin ME, Saltzberg MT, Jessup ML, Bart BA, Teerlink JR, et al Ultrafiltration versus intravenous diuretics for patients hospitalized for acute decompensated heart failure. J Am Coll Cardiol 2007;49:675–83. 10.1016/j.jacc.2006.07.073 17291932

[53] Bart BA, Goldsmith SR, Lee KL, Givertz MM, O’Connor CM, Bull DA, et al Ultrafiltration in decompensated heart failure with cardiorenal syndrome. N Engl J Med 2012;367:2296–304. 10.1056/NEJMoa1210357 23131078 PMC3690472

[54] Costanzo MR, Negoianu D, Jaski BE, Bart BA, Heywood JT, Anand IS, et al Aquapheresis versus intravenous diuretics and hospitalizations for heart failure. JACC Heart Fail 2016;4:95–105. 10.1016/j.jchf.2015.08.005 26519995

[55] McDonagh TA, Metra M, Adamo M, Baumbach A, Böhm M, Burri H, et al 2021 ESC guidelines for the diagnosis and treatment of acute and chronic heart failure. Eur Heart J 2021;42:3599–726. 10.1093/eurheartj/ehab368 34922348

[56] Aronson D, Nitzan Y, Petcherski S, Bravo E, Habib M, Burkhoff D, et al Enhancing sweat rate using a novel device for the treatment of congestion in heart failure. Circ Heart Fail 2023;16:E009787. 10.1161/CIRCHEARTFAILURE.122.009787 36321445

[57] Aronson D, Nitzan Y, Petcherski S, Shaul A, Abraham WT, Burkhoff D, et al Enhancing sweat rate for in-hospital and home-based decongestive therapy. J Card Fail 2025;32:5–12. 10.1016/j.cardfail.2025.01.010 39890012

[58] Rao VS, Ivey-Miranda JB, Cox ZL, Moreno-Villagomez J, Ramos-Mastache D, Neville D, et al Serial direct sodium removal in patients with heart failure and diuretic resistance. Eur J Heart Fail 2024;26:1215–30. 10.1002/ejhf.3196 38556717

[59] Caldwell JE, Ahonen E, Nousiainen U. Differential effects of sauna-, diuretic-, and exercise-induced hypohydration. J Appl Physiol Respir Environ Exerc Physiol 1984;57:1018–23. 10.1152/jappl.1984.57.4.1018 6501022

[60] Kihara T, Biro S, Imamura M, Yoshifuku S, Takasaki K, Ikeda Y, et al Repeated sauna treatment improves vascular endothelial and cardiac function in patients with chronic heart failure. J Am Coll Cardiol 2002;39:754–9. 10.1016/S0735-1097(01)01824-1 11869837

[61] Miyata M, Kihara T, Kubozono T, Ikeda Y, Shinsato T, Izumi T, et al Beneficial effects of Waon therapy on patients with chronic heart failure: results of a prospective multicenter study. J Cardiol 2008;52:79–85. 10.1016/j.jjcc.2008.07.009 18922381

[62] Källström M, Soveri I, Oldgren J, Laukkanen J, Ichiki T, Tei C, et al Effects of sauna bath on heart failure: a systematic review and meta-analysis. Clin Cardiol 2018;41:1491. 10.1002/clc.23077 30239008 PMC6489706

[63] Bekfani T, Behrendt T, Moebert R, Seibel S, Vargas KG, Abo Hwach A, et al The effect of intermittent hyperthermia (sauna) on functional capacity and quality of life in patients with heart failure and preserved ejection fraction: SAUNA-HFpEF. Eur Heart J 2025;46:ehaf784-1217. 10.1093/eurheartj/ehaf784.1217

[64] Trullàs JC, Morales-Rull JL, Casado J, Carrera-Izquierdo M, Sánchez-Marteles M, Conde-Martel A, et al Combining loop with thiazide diuretics for decompensated heart failure: the CLOROTIC trial. Eur Heart J 2023;44:411–21. 10.1093/eurheartj/ehac689 36423214

[65] Mullens W, Dauw J, Martens P, Verbrugge FH, Nijst P, Meekers E, et al Acetazolamide in acute decompensated heart failure with volume overload. N Engl J Med 2022;387:1185–95. 10.1056/NEJMoa2203094 36027559

[66] Mebazaa A, Davison B, Chioncel O, Cohen-Solal A, Diaz R, Filippatos G, et al Safety, tolerability and efficacy of up-titration of guideline-directed medical therapies for acute heart failure (STRONG-HF): a multinational, open-label, randomised, trial. Lancet 2022;400:1938–52. 10.1016/S0140-6736(22)02076-1 36356631

[67] Biegus J, Mebazaa A, Davison B, Cotter G, Edwards C, Čelutkienė J, et al Effects of rapid uptitration of neurohormonal blockade on effective, sustainable decongestion and outcomes in STRONG-HF. J Am Coll Cardiol 2024;84:323–36. 10.1016/j.jacc.2024.04.055 39019527

[68] Biegus J, Cotter G, Metra M, Ponikowski P. Decongestion in acute heart failure: is it time to change diuretic-centred paradigm? Eur J Heart Fail 2024;26:2094–106. 10.1002/ejhf.3423 39169731

